# Progress in Surgery

**Published:** 1899-12-09

**Authors:** 


### Progress in Surgery.

### SURGERY OF THE SPINE.

**Differentiation of Vertebral Syphilis from Potts' Disease of the Spine.**—Dr. Chas. W. Burr[1] reports the case of a gentleman, aged 31, who complained of loss of power in the legs and arms. He had suffered from syphilis, and had sustained two severe accidents, neither of which, however, was followed by spinal symptoms. Ten months before being seen he began to notice a retraction of his head and stiffness in the neck. Seven months after he lost the power of the left arm, left leg, right arm and right leg, in that order, and had difficulty in micturition. He was free of pain. On examination a firm, ill-defined swelling was to be made out on either side of the cervical vertebræ. It was apparently attached to the bones and was painless, although tender to pressure. There was no evidence of a retro-pharyngeal abscess. There were hyperæsthetic spots over the body, which shifted their position from day to day, and he had no true appreciation of temperature. Other nervous symptoms were present. He was treated by iodide of potassium and mercury, and after several months' treatment the deformity of the spine had disappeared, and his motor and sensory symptoms were all but gone.

He obviously suffered from syphilitic meningomyelitis, the swelling in the neck being a gumma. The diagnosis lay between this and a malignant growth or tuberculous disease.

In commenting on this case, Dr. Burr remarked that when a well-marked swelling is externally visible and palpable there is no difficulty in diagnosis; whereas when the growth projects towards the spinal canal and presses on the cord it is almost impossible. The bodies are rarely affected by syphilis, but when they are the symptoms closely simulate tuberculous disease. Zasinski, who has collected a large number of cases of syphilitic spinal disease, referring to the differential diagnosis, concludes that primary tuberculosis of the spine does not occur in the adult, and that therefore if angular curvature and other symptoms suggestive of Potts' disease be present, in the absence of visceral tuberculosis, the diagnosis lies between tumour and syphilis. Burr, Davies, and Taylor do not agree with this statement, as all have seen cases of primary tuberculosis of the spine apart from visceral lesions. Leyden lays stress on the fact that syphilitic lesions of the spine are often multiple and may attack any portion of the vertebræ, whereas tuberculous lesions are usually single and confined to the bodies or intervertebral discs. Tuberculous lesions are therefore usually motor, being confined to the anterior portion of the cord, and are usually symmetrical, while syphilitis affects any portion of the periphery of the cord and gives rise to unilateral or other aberrant forms of symptoms.

**Non-Tuberculous Inflammation of the Spine.**—In a recent paper Dr. Halsted Myers[2] expresses his doubt as to the primary occurrence of tuberculosis in the spine. In all the autopsies he has made he has found other tuberculous lesions, in glands or elsewhere.

[1] Annals of Surgery, June, 1899. [2] New York Med. News, No. 19, 1892.

